# The bending of cell sheets - from folding to rolling

**DOI:** 10.1186/1741-7007-9-90

**Published:** 2011-12-29

**Authors:** Ray Keller, David Shook

**Affiliations:** 1 Department of Biology, 241 Gilmer Hall, University of Virginia, Charlottesville, VA 22904, USA

## Abstract

The bending of cell sheets plays a major role in multicellular embryonic morphogenesis. Recent advances are leading to a deeper understanding of how the biophysical properties and the force-producing behaviors of cells are regulated, and how these forces are integrated across cell sheets during bending. We review work that shows that the dynamic balance of apical versus basolateral cortical tension controls specific aspects of invagination of epithelial sheets, and recent evidence that tissue expansion by growth contributes to neural retinal invagination in a stem cell-derived, self-organizing system. Of special interest is the detailed analysis of the type B inversion in *Volvox *reported in *BMC Biology *by Höhn and Hallmann, as this is a system that promises to be particularly instructive in understanding morphogenesis of any monolayered spheroid system.

See research article: http://www.biomedcentral.com/1741-7007/9/89

## Commentary

Cell sheet bending is an active process, required for normal morphogenesis in many instances of multicellular embryogenesis, including the formation of the germ layers during gastrulation, the gut and neural tube, the eye, the otic system and the diverticula of the gut. A number of mechanisms have been proposed for cell sheet bending, including growth pressure, cell shape changes driven by cell-cell or cell-matrix adhesion, or by the cytoskeleton, for example, each with varying levels of experimental support. Recent combinations of live imaging, molecular interdictions, biophysical analysis, and computational modeling are providing a much better understanding of the key biomechanical processes underlying how cells generate forces, how local forces are integrated over large cell sheets, and how morphogenic function depends on geometric and biomechanical context.

## Cell wedging - balancing cellular tensions

**Figure 1:**
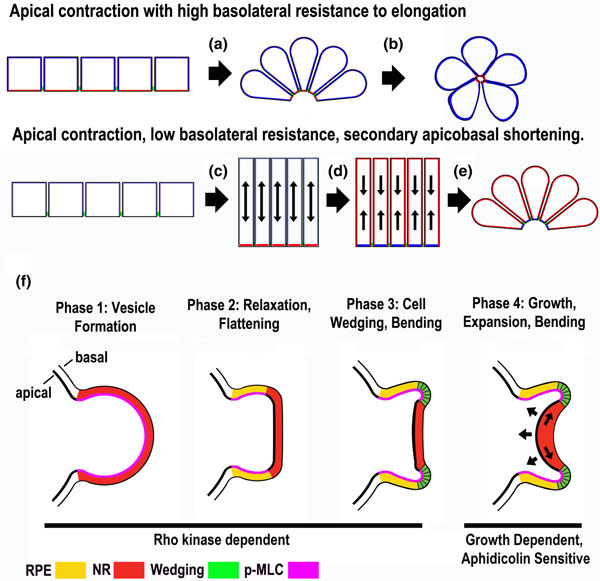
**Sectional diagrams showing modes of epithelial bending**. During monophasic epitheilial bending apical contraction occurs with relatively high basolateral resistance to cell elongation **(a,b)**. In the biphasic mode, apical contraction occurs in the presence of relatively low resistance to elongation **(c)**; this is followed by a second phase of apical-basal shortening in the presence of contracted apices, which results in bending **(d,e) **[2,3,5]. **(f) **Sectional diagrams illustrating bending of the neural retina epithelium in a stem cell-derived mammalian optic vesicle in culture [6]. RPE = retinal pigment epithelium; NR = neural retina; p-MLC = phosphorylated myosin light chain.

One of the most studied mechanisms of bending a cell sheet is the 'wedging' of individual epithelial cells by contraction of their apical ends driven by myosin motor proteins acting on actin filaments (actomyosin-mediated contraction). The apical ends of these cells are mechanically linked to one another by adherens junctions, which integrate these locally generated apical constriction forces over the entire sheet, causing it to bend (Figure 1, steps a to e). Apical constriction does not act alone, however, as recent work shows that the dynamic balance of cortical tension in apical versus basolateral cell domains plays a large role in regulating the specific aspects of cell shape, such as apical-basal elongation, that determine how the sheet is bent. In some systems, such as in the formation of the 'bottle-shaped' cells at the site of blastopore invagination in amphibians, bending of the cell sheet is monophasic. With apical constriction, cytoplasm is forced basally but is simultaneously met with apical-basal resistance to elongation, thereby producing cell wedging, and bending of the sheet in one step [1,2] (Figure 1, step a). If cut free of the mechanical load of surrounding tissues, the bottle cells quickly shorten further, becoming nearly spherical, and bending the sheet to the extreme (Figure 1, step b), suggesting that resistance to apical-basal elongation during apical constriction is an integral component of the wedging process [1]. In other systems, including ventral furrow (mesoderm) invagination in *Drosophila *[3] and endoderm invagination in ascidians [4], cell sheet bending is biphasic. During apical contraction, movement of cytoplasm basally is met with less, or no resistance, and the cells first elongate (Figure 1, step c); then, in the second phase, an apical-basal contraction shortens the cells, forces cytoplasm basally, which in the presence of the constricted apices generates wedge-shaped cells and bending of the sheet [4] (Figure 1, steps d and e).

Recent work using live imaging, microsurgical cell ablations, and computational modeling shows that the progression of the biphasic invagination of the endoderm during ascidian gastrulation is determined by sequential actomyosin-mediated contractions of apical and basolateral domains of the endodermal cells [4]. A Rho/Rho kinase-dependent enrichment of monophosphorylated, and therefore activated, myosin (1p-myosin) across the apical cortex of the cells results in apical contraction, and the cells elongate without invaginating (Figure 1, step c). In the second phase, a Rho/Rho kinase-independent enrichment of 1p-myosin in the basolateral domain is essential for shortening and rounding of the cells, known as 'collared rounding', which produces wedging and invagination (Figure 1, steps d and e), provided that apical expansion is prevented by a Rho/Rho kinase-dependent increase of 2p-myosin in the peripheral regions of the already contracted apices (blue region in the cells before and after step 'e' of Figure 1). Further analysis of how the assembly and regulation of the actin-myosin cytoskeleton in apical versus basolateral cortical domains controls the dynamic balance of cortical tension should establish the biophysical basis of this mode, as well as the monophasic mode of bending.

## Combining mechanisms - cell wedging, regulated tissue stiffness, and growth

Other folding events, perhaps most of them, are composites of several mechanisms, and involve both local cell level and global tissue level biomechanical interactions [1,2,5]. Embryonic stem cell-derived neuroepithelial vesicles can form optic cups, mimicking those of mouse embryos, in a self-organizing manner, in culture, without interactions with other tissues [6] (Figure 1f). Multiphoton live imaging reveals four phases in the process. In phase one, a hemispherical bulge of columnar, monolayered epithelial cells extends from the neuroepithelial vesicle; this bulge contains high levels of phosphorylated myosin light chain (pMLC) and is stiffened compared to other parts of the tissue, as assayed by atomic force microscopy (AFM), which is able to measure force in tissues. In phase two, the distal part of the bulge (the differentiating neural retina) has decreased levels of pMLC and increased flexibility, and it flattens. In phase three, the margin of the flattened retinal epithelial region shows elevated apical pMLC, high stiffness, apical constriction, and cell wedging, which bends the less stiff retinal epithelium inward. In phase four, the flexible retinal epithelium grows, and its tangential expansion against the stiff, restraining retinal pigmented epithelium results in inward buckling, largely mimicking normal, *in vivo *optic cup formation (Figure 1f). The first three phases require Rho kinase and the actomyosin cytoskeleton; phase four does not but is sensitive to aphidicolin, which inhibits DNA synthesis, and thus cell division and growth. These experiments again highlight the role of actomyosin regulation of tissue stiffness and contraction, the role of local cell wedging in biasing the outcome of subsequent, large-scale mechanical interactions between stiff and flexible regions, and the role of growth in bending cell sheets.

## From folding to rolling: lessons from *Volvox*

**Figure 2:**
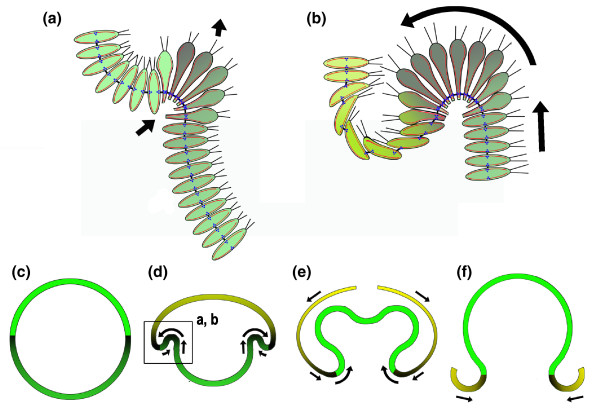
**A sectional diagram illustrating aspects of the type B inversion in *Volvox***. **(a,b) **The initial steps in forming the subequatorial zone of cell wedging that progresses posteriorly and rolls or involutes (curved arrow) the posterior half into the anterior half are shown. **(c-f) **The location of this event in the context of the entire inversion is shown (box in **(d)**). The direction of the progression of zones of cell shape change is indicated by shading from dark to light (see text for discussion). Red, microtubules; blue, kinesins; black, cytoplasmic bridges. Based on [7-9].

*Volvox*, a multicellular algae, takes the process of bending of cell sheets to a new level - it uses bending to turn the entire organism inside out. *Volvox *embryos consist of hollow, monolayered spheroids of cells joined by cytoplasmic bridges (CBs), a product of incomplete cytokinesis. They initially have their future outer, biflagellated ends facing inward and achieve their mature morphology by inverting [7]. This inversion can occur in two ways, known as type A and type B inversions. In type A inversion, a zone of transient cell wedging, narrowed ends outward, forms at the edge of a hole, the phialopore, at the anterior end of the embryo. This zone then progresses posteriorly, and curls, or rolls the embryo inside out in one step [7]. Cell wedging depends on a cortical microtubule array, which transforms the spindle-shaped cells into elongated, tapered cells. Alone, this produces only minimal bending of the sheet, but in addition the cells are moved relative to the CBs, such that the CBs come to lie at the outer, tapered ends of the cells by a microtubule/kinesin-dependent mechanism [7,8]. This moves the fulcrum point of the 'wedges' to the most effective position to produce acute bending (Figure 2a).

The lesser-known type B inversion has now been analyzed in detail in *Volvox globator *by Höhn and Hallmann [9]. It differs from type A inversion in major ways that make it a prime model system for understanding the general cell biological and biomechanical principles of bending sheets, particularly in spheroid systems, but also of likely application to vertebrate model systems. In type B inversion, a similar circular zone of cell wedging is initiated (Figure 2a), but just below the equator of the embryo where it generates a circular crease of acute, inward lifting of the posterior half relative to the anterior half (Figure 2b,c). This circular wave of transient wedging progresses posteriorly (Figure 2c,d) [9] and rolls the inside of the posterior half progressively against the inside surface of the anterior half (Figure 2c,d), after which the wedge-shape cells adopt a pencil shape with the CBs at the same end, now the inner face of the cells. As the posterior half rolls into the anterior half, the cells of the anterior half adopt a flattened, discoid shape and become arrayed in a shingled fashion in which the CBs appear asymmetric across the cells (Figure 2b). This change progresses anteriorly from its equatorial origin at the original crease (Figure 2b,e,f). As the posterior half reaches the inside of the anterior pole, a hole forms in the latter, and the anterior half begins to slide down the side of the involuted posterior half (Figure 2e,f); the discoid cells at the posterior margin of the un-involuted anterior half, progressively, and in an equator-to-anterior order, adopt a pencil shape [9].

Of special interest is the well-defined, progressive zone of transient cell wedging that produces an 'involution', which is when a sheet is rolled around an inflection point, much like the rolling of a bulldozer track around its wheels [2]. Many tissue movements in biomedical model systems show involution, either alone or as part of an invagination, such as when the sheet of cells both bends to form an inflexion zone and also rolls over it. Many of these involutions occur in spheroid or cylindrical contexts, but few of these systems offer the analytical advantages of *Volvox *inversion, particularly type B, where the rolling bends are robust, fast, relatively free of complex linkages to other processes, and in a system that should be amendable to experimental manipulation and computational modeling. The consequences of local cell behaviors in spheroid systems have not been sufficiently explored, although computational modeling shows that they are important and can drive invagination, for example, in counter-intuitive ways [5]. The type B inversion is a particularly rich system for investigating the biophysical basis of these movements and the signaling underlying them [9]. It is also well-suited to investigation of their evolution, given that one type of inversion appears to have evolved independently of the other three or four times within the volvocine multicellular algae, consistently in conjunction with changes in reproductive strategies and retention of CBs between embryos and adults prior to inversion (see [10] and [9]). These findings from *Volvox *are likely to be particularly instructive for other model systems.
